# An Fc-Optimized CD133 Antibody for Induction of Natural Killer Cell Reactivity Against Colorectal Cancer

**DOI:** 10.3390/cancers11060789

**Published:** 2019-06-07

**Authors:** Bastian J. Schmied, Fabian Riegg, Latifa Zekri, Ludger Grosse-Hovest, Hans-Jörg Bühring, Gundram Jung, Helmut R. Salih

**Affiliations:** 1 Clinical Collaboration Unit Translational Immunology, German Cancer Consortium (DKTK) and German Cancer Research Center (DKFZ), Partner site, 72076 Tuebingen, Germany; Bastian.Schmied@med.uni-tuebingen.de (B.J.S.); Fabian.Riegg@med.uni-tuebingen.de (F.R.); l.zekri-metref@dkfz.de (L.Z.); 2 DFG Cluster of Excellence 2180 “Image-guided and Functional Instructed Tumor Therapy (iFIT)”, 72076 Tuebingen, Germany; 3 Department for Immunology, Eberhard Karls University, 72076 Tuebingen, Germany; grosse-hovest@synimmune.de (L.G.-H.); gundram.jung@uni-tuebingen.de (G.J.); 4 Department of Hematology and Oncology, Eberhard Karls University, 72076 Tuebingen, Germany; hans-joerg.buehring@uni-tuebingen.de

**Keywords:** colorectal cancer, immunotherapy, antibody, NK cells, ADCC, CD133, prominin-1

## Abstract

The introduction of monoclonal antibodies (mAbs) has largely improved treatment options for cancer patients. The ability of antitumor mAbs to elicit antibody-dependent cellular cytotoxicity (ADCC) contributes to a large extent to their therapeutic efficacy. Many efforts accordingly aim to improve this important function by engineering mAbs with Fc parts that display enhanced affinity to the Fc receptor CD16 expressed, e.g., on natural killer (NK) cells. Here we characterized the CD133 mAb 293C3-SDIE that contains an engineered Fc part modified by the amino acid exchanges S239D/I332E—that reportedly increase the affinity to CD16—with regard to its ability to induce NK reactivity against colorectal cancer (CRC). 293C3-SDIE was found to be a stable protein with favorable binding characteristics achieving saturating binding to CRC cells at concentrations of approximately 1 µg/mL. While not directly affecting CRC cell growth and viability, 293C3-SDIE potently induced NK cell activation, degranulation, secretion of Interferon-γ, as well as ADCC resulting in potent lysis of CRC cell lines. Based on the preclinical characterization presented in this study and the available data indicating that CD133 is broadly expressed in CRC and represents a negative prognostic marker, we conclude that 293C3-SDIE constitutes a promising therapeutic agent for the treatment of CRC and thus warrants clinical evaluation.

## 1. Introduction

The introduction of immunotherapy to induce a specific antitumor immune response constitutes—as monotherapy or combinatorial treatment—A well established option for cancer treatment [1]. Especially monoclonal antibodies (mAbs) have largely improved the treatment options for patients with malignant disease. For example, rituximab and trastuzumab are widely used for therapy of patients with B-cell non-Hodgkin´s lymphoma and human epidermal growth factor receptor 2 (HER2)-positive breast cancer, respectively [2,3]. However, despite their undisputed success, the therapeutic efficacy of these and other antitumor mAbs is still far from satisfactory. While various factors influence the susceptibility of tumor cells to therapeutic mAbs (such as mutant forms of receptors, alternative signaling pathways, genetic variability and receptor shedding; e.g., [4]), one approach to improve efficacy is to enhance the immunostimulatory potency of antitumor mAbs [5]. With regard to mAbs that target tumor cells and mediate their effects (at least in part) via induction of antibody-dependent cellular cytotoxicity (ADCC), one strategy is to modify their Fc part in order to enhance the affinity to the Fc receptor CD16. This improves the recruitment of CD16 expressing immune cells, among which, at least in humans, natural killer (NK) cells are particularly important to induce ADCC [6,7]. The latter constitutes one of the major effector mechanisms by which antitumor mAbs mediate their beneficial effects, at least in hematopoietic malignancies (e.g., [8]). To improve ADCC, Fc parts can be modified either with regard to their glycosylation patterns or by changes in the amino acid sequence. Glyco-optimized mAbs, like obinituzumab, have been approved by the FDA, and others as well as antibodies with Fc parts carrying amino acid substitutions (e.g., S239D/I332E (SDIE) [9]) are currently being evaluated in clinical trials [5].

Recently we reported on the preclinical characterization of Fc-optimized mAbs and antibody-like constructs carrying the SDIE modification for immunotherapy of leukemia [10,11,12,13,14]. Besides an Fc-optimized FLT3 (CD135) mAb that is presently undergoing clinical evaluation (ClinicalTrials.gov ID NCT02789254), this comprised a construct targeting CD133 (prominin-1). The latter is a pentaspan transmembrane glycoprotein and, beyond leukemia, also expressed in various solid tumors [15]. While CD133 has been implicated to play a role, e.g., in chemotherapy resistance and metastasis, its exact biological function remains to be fully elucidated [16]. Particularly in colorectal cancer (CRC), CD133 is frequently expressed and constitutes a negative prognostic marker [17,18,19]. Since so far immunotherapeutic options for CRC treatment are rather limited, with applications of anti-epidermal growth factor receptor (EGFR) mAbs and checkpoint inhibitors being restricted to the subsets of patients with metastatic disease that display rat sarcoma (RAS) wildtype and microsatellite instability-high/DNA mismatch repair deficiency, respectively, we here set out to evaluate 293C3-SDIE as a potential immunotherapeutic option for CRC. 

## 2. Results

### 2.1. Binding of Different CD133 mAbs to CRC Cells

**Figure 1:**
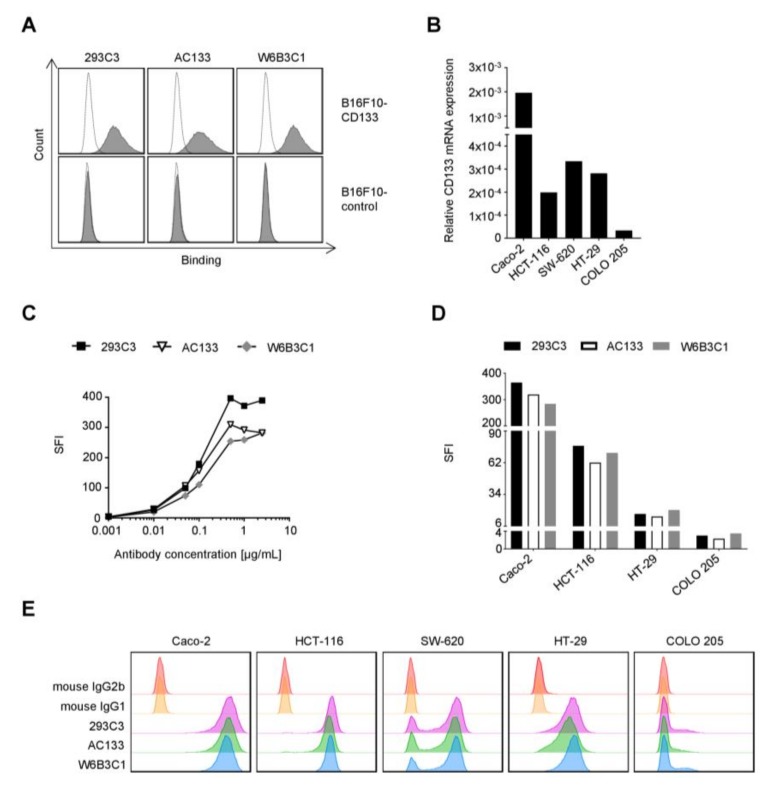
Comparative binding analysis of different CD133 monoclonal antibodies (mAbs) to colorectal cancer (CRC) cells. (**A**) Specific binding of three different commercially available CD133 mAbs (293C3, AC133, and W6B3C1) was determined by flow cytometry using B16F10-CD133 and B16F10-control transfectants. Cells were incubated with 5 µg/mL CD133 mAb followed by a goat anti-mouse phycoerythrin (PE) conjugate. Shaded peaks: CD133 mAbs; open peaks: controls. (**B**) Relative CD133 mRNA expression in five CRC cell lines—which were used as a model for mAb binding analyses—was determined by quantitative PCR as described in the method section. (**C**) The CD133 mAb surface binding was comparatively analyzed in flow cytometry experiments by incubating CD133 mRNA^high^ Caco-2 cells with increasing mAb concentrations of the different CD133 mAbs or isotype controls followed by a goat anti-mouse PE conjugate. Specific fluorescence intensity (SFI) levels were calculated as described in the method section. (**D**,**E**) The CD133 mAb binding to the panel of five CRC cell lines was comparatively analyzed by flow cytometry. Cells were incubated with 1 µg/mL CD133 mAb or isotype controls followed by a goat anti-mouse PE conjugate. Specific fluorescence intensity (SFI) levels (not applicable for SW-620 cells due to bimodal CD133 expression) and histograms are depicted in (**D**,**E**), respectively. Representative data of one experiment from a total of at least two with similar results is shown.

Recently we observed pronounced differences in the binding of three different mouse anti-human CD133 mAbs to acute myeloid leukemia (AML) cells. Based on superior binding characteristics, the clone 293C3 was accordingly chosen for construction of our therapeutic construct 293C3-SDIE [14]. To determine whether differential reactivity also occurs in CRC, we first confirmed specific binding using CD133 or control transfected B16F10 cells (Figure 1A). The mouse background of these cells served to exclude that (CD133) cross-reactivity of any of the three anti-human CD133 mAb clones influenced our results. Subsequently, we compared the binding of clone (i) 293C3, (ii) AC133, which is used in other CD133 targeting cancer therapeutics [20], and (iii) W6B3C1 using a panel of five different CRC cell lines that reportedly express CD133 [21,22,23]. CD133 mRNA expression was observed by quantitative PCR in all CRC cell lines, but with profoundly different levels (Figure 1B). Caco-2 cells, which displayed the highest CD133 mRNA levels, were then employed in dose titration experiments with all three antibodies. Flow cytometric analysis revealed that all three clones achieved saturated binding at approximately 1 µg/mL (Figure 1C). This concentration was then used to comparatively analyze binding in the panel of the five CRC cell lines with different biological characteristics (Table 1; [24,25]). As shown in Figure 1D,E, no marked differences with regard to binding were observed with the different CD133 mAb clones. While this was in contrast to our findings in AML, where clone 293C3 was superior to the other clones [14], and the reason for this discrepancy so far remain elusive, these results warranted the use of our therapeutic construct 293C3-SDIE in further analyses.

### 2.2. Generation and Characterization of 293C3-SDIE in CRC

**Figure 2:**
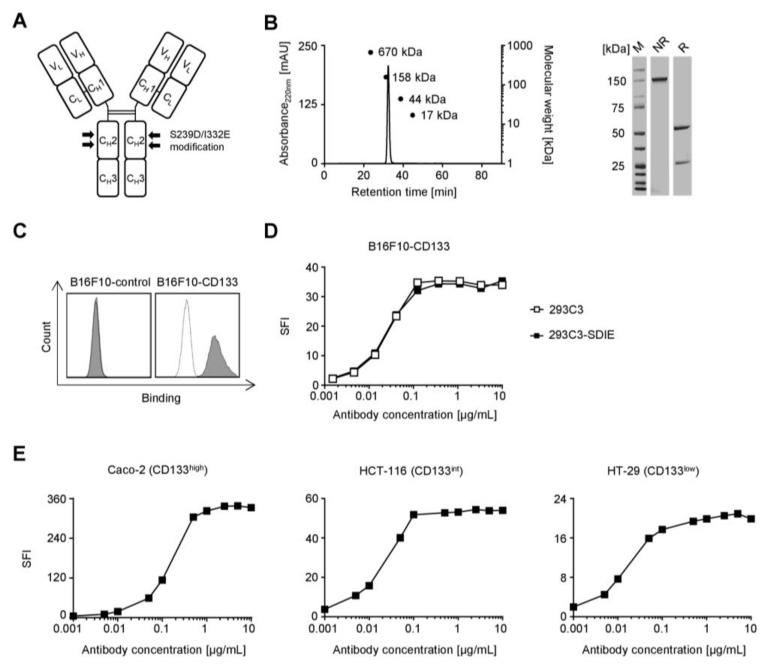
Generation and characterization of 293C3-SDIE in colorectal cancer (CRC). (**A**) Schematic illustration of 293C3-SDIE. (**B**) Purified 293C3-SDIE was analyzed by size exclusion chromatography (left) and SDS-PAGE (right). mAU: milli absorption unit; R: reduced; NR: non-reduced; M: marker. (**C**) B16F10-CD133 and B16F10-control transfectants were incubated with 10 µg/mL 293C3-SDIE or Iso-SDIE followed by an anti-human phycoerythrin (PE) conjugate. Shaded peaks: 293C3-SDIE; open peaks: Iso-SDIE. (**D**) B16F10-CD133 transfectants were incubated with the indicated concentrations of 293C3 or 293C3-SDIE and their respective isotype controls followed by an anti-mouse or anti-human PE conjugate. (**E**) The colorectal cancer (CRC) cell lines Caco-2 (CD133^high^: left), HCT-116 (CD133^int^: middle), and HT-29 (CD133^low^: right) were incubated with the indicated concentrations of 293C3-SDIE or Iso-SDIE followed by an anti-human PE conjugate. Int: intermediate; SFI: specific fluorescence intensity.

As previously described [14], mAb clone 293C3 was chimerized (backbone: human immunoglobulin G1/K constant region) and Fc-optimized by introducing the S239D/I332E modification in the constant heavy chain domain 2 (CH2) which is illustrated in Figure 2A. An Fc-optimized control protein with irrelevant target specificity termed Iso-SDIE served as control. Upon production as described in the method section, 293C3-SDIE was obtained with good yields, and analysis by sodium dodecyl sulfate–polyacrylamide gel electrophoresis (SDS-PAGE) and gel filtration revealed the expected molecular weights of ~24, ~50, and ~148 kDa for light chain (LC), heavy chain (HC), and full mAb, respectively, and confirmed the lack of aggregates (Figure 2B). Flow cytometric analyses including dose titrations with B16F10 transfectants confirmed that the chimerization and Fc-optimization process had not affected the specificity and affinity of 293C3-SDIE as compared to the parental murine mAb (Figure 2C,D). Next we performed dose titration experiments using three CRC cell lines with high, intermediate, and low CD133 surface antigen densities (Caco-2, CD133^high^; HCT-116, CD133^int^; HT-29, CD133^low^). Flow cytometry revealed that saturating doses positively correlated with CD133 surface levels, but in all cases 1 µg/mL was sufficient to achieve saturating binding (Figure 2E). 

### 2.3. Direct Effects of 293C3-SDIE on CRC Cell Viability

**Figure 3:**
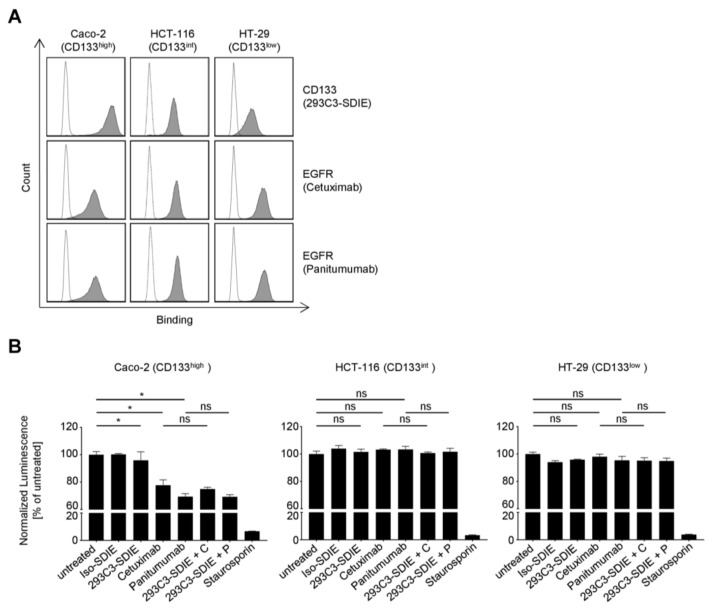
Direct effects of 293C3-SDIE on colorectal cancer (CRC) cell viability. (**A**) CD133 and epidermal growth factor receptor (EGFR) expression on CRC cell lines were comparatively analyzed by flow cytometry using 293C3-SDIE, cetuximab and panitumumab and their respective isotype controls (all at 1 µg/mL) followed by an anti-human phycoerythrin (PE) conjugate. Shaded peaks: specific mAbs; open peaks: isotype controls. (**B**) Caco-2 (left), HCT-116 (middle), and HT-29 (right) cells were incubated with 1 µg/mL of the indicated mAbs for three days. ATP levels were then determined by CellTiterGlo assays. Representative data of one experiment from a total of at least two with similar results are shown. C: cetuximab; int: intermediate; ns: not significant; P: panitumumab; *: significant (*p*-value < 0.05).

As CD133 was previously suggested to be involved in tumor cell survival and proliferation and CD133 mAb binding could have an influence in this context [16,26], we next determined whether 293C3-SDIE directly affected tumor cell viability. To this end, the CRC cell lines Caco-2, HCT-116, and HT-29 with high, intermediate, and low CD133 antigen densities, respectively, were incubated with 293C3-SDIE in the absence of immune effector cells. Notably, all these cell lines also express EGFR, which is therapeutically targeted by cetuximab and panitumumab (Figure 3A), and these two mAbs were included in the analysis. Analysis of ATP levels as surrogate marker for the amount of viable cells revealed that 293C3-SDIE had no effect (Figure 3B). While two of the CRC cell lines were not responsive to the EGFR blockade, the viability of Caco-2 cells was clearly reduced by the anti-EGFR mAbs. This differential reactivity is in line with data previously published by other investigators [27]. Since CD133 can interact with EGFR and has been hypothesized to contribute to resistance to EGFR-targeting drugs [16], we also determined whether simultaneous targeting of CD133 by 293C3-SDIE would sensitize CRC cells to anti-EGFR mAb treatment. To this end, CRC cells were incubated with either cetuximab or panitumumab alone or in combination with 293C3-SDIE, but anti-EGFR mAbs treatment effects were not further increased by 293C3-SDIE. 

### 2.4. Induction of NK Cell Reactivity Against CRC Cells by 293C3-SDIE

**Figure 4:**
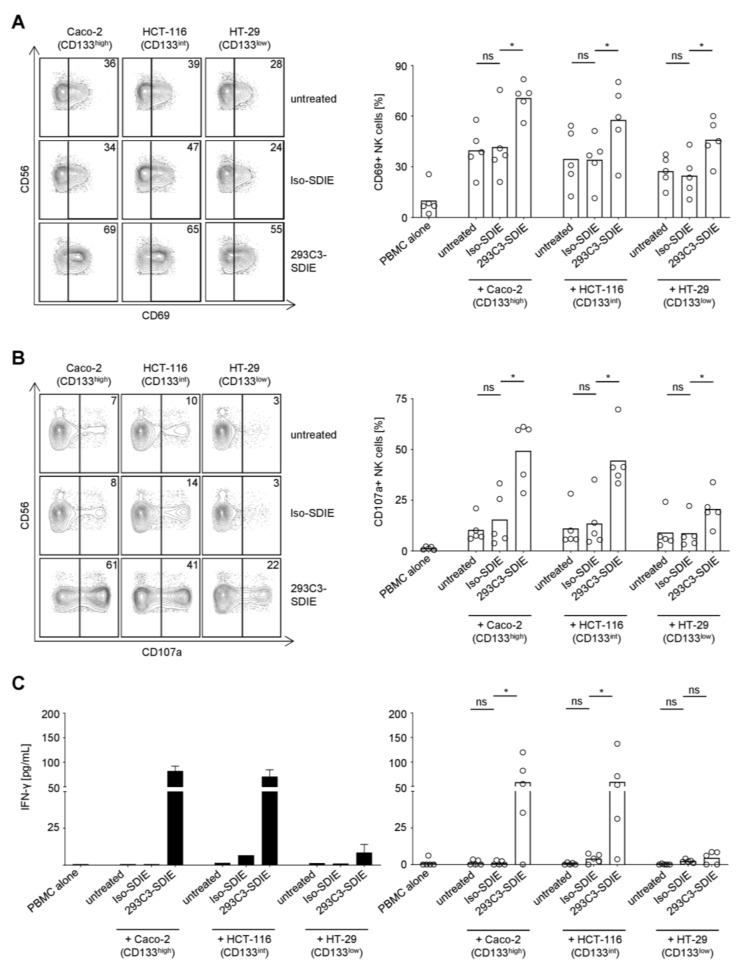
Induction of natural killer (NK) cell activity by 293C3-SDIE in the presence of colorectal cancer (CRC) cells. Peripheral blood mononuclear cells (PBMC) of healthy donors were cultured with or without the indicated CRC cell lines at an effector to target ratio of 2.5:1 in the presence or absence of 293C3-SDIE/Iso-SDIE (1 µg/mL). On the left, exemplary results obtained in a single experiment with PBMC of a single donor are shown; right panels depict combined analyses of data obtained with PBMC from five independent donors (bars represent respective means). (**A**) Activation of NK cells identified as CD14-CD56+CD3- cells within PBMC was determined after 24 h by flow cytometry for CD69. (**B**) Cells were cultured for 4 h in the presence of anti-human CD107a-PE/GolgiStop/GolgiPlug and NK cells subsequently analyzed by flow cytometry for CD107a as surrogate marker for degranulation. (**C**) Cells were cultured for 6 h before supernatants were analyzed for Interferon (IFN)-γ by an enzyme-linked immunosorbent assay. Int: intermediate; ns: not significant; *: significant (*p*-value < 0.05).

Next we determined how 293C3-SDIE induced NK cell mediated anti-tumor immunity against CRC cells. To this end, peripheral blood mononuclear cells (PBMCs) of healthy donors containing NK cells as effector cells were cultured with the CRC cell lines Caco-2, HCT-116, and HT-29 with their high, intermediate, and low CD133 antigen densities, respectively, in the presence or absence of 293C3-SDIE or isotype control. Flow cytometric analysis of CD69 on NK cells revealed that 293C3-SDIE profoundly induced NK cell activation, while the control mAb with irrelevant target specificity had no effect (Figure 4A). In line, 293C3-SDIE specifically induced NK cell degranulation as revealed by flow cytometric detection of CD107a (Figure 4B). Additionally, the NK cell release of Interferon (IFN)-γ, an immunomodulatory cytokine that elicits direct anti-tumor effects and by which NK cells shape subsequent adaptive immune responses, was specifically induced by 293C3-SDIE (Figure 4C). Notably, in all cases the observed 293C3-SDIE effects positively correlated with antigen density. 

**Figure 5:**
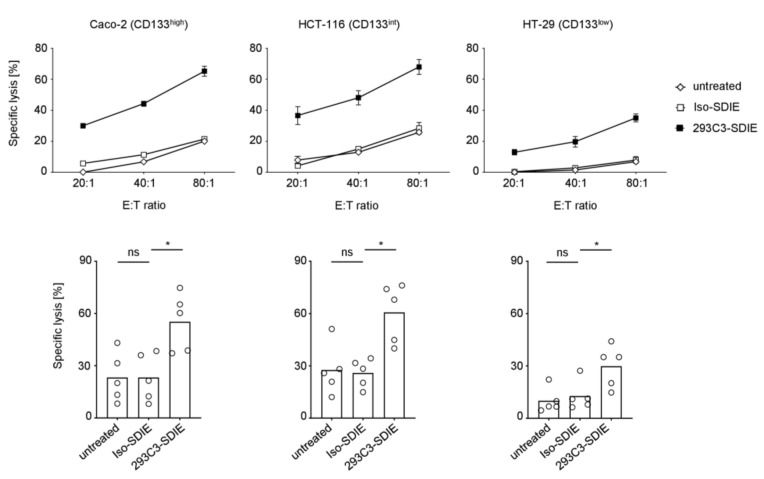
Induction of natural killer (NK) cell mediated colorectal cancer (CRC) cell lysis by 293C3-SDIE. Peripheral blood mononuclear cells (PBMC) of healthy donors were cultured with Caco-2 (left), HCT-116 (middle), and HT-29 cells (right) in the presence or absence of 293C3-SDIE/Iso-SDIE (1 µg/mL). Tumor cell lysis was measured after 2 h by Europium based cytotoxicity assays. In the top exemplary data over a broad range of effector to target (E:T) ratios with PBMC of one donor and in the bottom pooled data (bars represent respective means) at an E:T ratio of 80:1 with PBMC of five different donors are shown. Int: intermediate; ns: not significant; *: significant (*p*-value < 0.05).

Finally we examined whether the above-described effects on NK cell activity were mirrored by ADCC and a resulting tumor cell lysis. Europium based cytotoxicity assays revealed that treatment with 293C3-SDIE induced a clearly target-antigen restricted lysis, and this was observed with all tested cell lines (Figure 5). In line with the results observed for NK cell activity, lysis rates again positively correlated with CD133 antigen density on CRC target cells. Thus, 293C3-SDIE is capable to potently stimulate NK cell immunity against CRC cells. Furthermore, it is of particular interest that 293C3-SDIE was able to induce anti-tumor immunity against microsatellite stabile (Caco-2, HT-29) and RAS-mutated (HCT-116) CRC forms, where checkpoint blockade and anti-EGFR mAbs were found to lack efficiency in patients, respectively.

## 3. Discussion

In the present study, we report on the preclinical characterization of 293C3-SDIE for treatment of CRC. 293C3-SDIE is a chimerized and Fc-optimized CD133 mAb recently introduced for induction of NK cell ADCC against leukemia. Evaluation in CRC appeared rational since CD133 is highly expressed in solid tumors, particularly in CRC, where it constitutes a negative prognostic marker, and immunotherapeutic options so far are rather limited. Our analyses revealed that 293C3-SDIE is well suited to target CD133 expressing CRC cells for NK cell ADCC because 293C3-SDIE showed convincing binding characteristics in CRC and potently induced anti-tumor immunity as determined in multiple experimental settings using CRC cell lines and NK cells contained in PBMC from healthy donors as effector cells. 

NK cells belong to the group of cytotoxic lymphocytes and not only exert functions in innate immunity, but also influence adaptive immune responses [28]. They largely contribute to cancer immunosurveillance; thus, multiple efforts presently aim to engraft NK cells in cancer treatment [29]. A well established approach to achieve this goal is the application of antitumor antibodies to induce ADCC, as highlighted by the success, e.g., of rituximab, which is established for the treatment of B cell malignancies and the efficacy of which is largely based on ADCC [8]. While other immune cells, e.g., monocytes, also express CD16, it is firmly established that in humans it is NK cells that mediate this important antibody function [6,7]. At present, multiple strategies aim to further increase ADCC by generating Fc-optimized antitumor mAbs with enhanced affinity to CD16. Besides by modifications of the glycosylation pattern, this can be achieved by amino acid modifications such as the substitutions S239D/I332E (SDIE modification) in the Fc part’s CH2 domain contained in 293C3-SDIE. Notably, many other Fc-optimized mAbs that currently undergo clinical evaluation, e.g., MOR00208 (anti-CD19; ClinicalTrials.gov ID: NCT01685021), margetuximab (anti-HER2; NCT01828021), FLYSYN (anti-FLT3; NCT02789254), MEN1112 (anti-CD157; NCT02353143) and BI 836858 (anti-CD33; NCT02240706, NCT03013998), comprise the SDIE modification.

After evaluating 293C3-SDIE for treatment of leukemia, we reasoned that CD133 would also constitute a promising target antigen for an Fc-optimized antibody in CRC. So far, established antibody-based approaches in CRC are restricted to a minority of patients only. Cetuximab and panitumumab are approved for treatment of patients with metastatic disease and only for patients with wildtype RAS accounting for 44% of metastatic CRC patients [30]. Immune checkpoint blockade is only available for CRC patients with metastasized disease and microsatellite instability-high/DNA mismatch repair deficiency, which accounts for ~5% of metastatic CRC patients [31]. CD133 has been suggested to be involved, amongst others, in chemotherapy resistance and metastasis, and was found to constitute a negative prognostic marker in CRC as shown in two meta-analyses [16,18,19]. CD133 is further expressed in a high number of CRC cases [17,18], which constitutes an important prerequisite for a therapeutic target antigen. It is thus not surprising that presently various CD133 targeting immunotherapeutics are under development, which, beyond 293C3-SDIE, comprises immunotoxins, CAR-T cells, bi-/tri-/tetraspecific mAbs, nanoparticles, adaptamers, and dendritic cell (DC)-based vaccination strategies. While most of these approaches aim to stimulate antitumor immunity against CD133-expressing target cells, they differ largely in many aspects, including, among others, the efforts required for production and the associated “costs of goods” and, importantly, efficacy and potential side effects. 

While substantial further preclinical work and results of clinical studies are required to decide on the finally optimal CD133-targeting strategy, our findings demonstrate that 293C3-SDIE is produced well and with only minor aggregation tendency. This is in contrast to more artificial constructs such as the bispecific T cell engager (BiTE) format, where aggregates can cause unspecific T cell activation. In addition, 293C3-SDIE would constitute a “ready-to-use,” off the shelf product, which would avoid the delay of treatment (about three weeks) that is required for the production of CAR-T cells and contributes to their vast costs upon clinical application. Furthermore, the concentration of 1 µg/mL 293C3-SDIE that was found to be sufficient to saturate CD133 binding and to potently induce ADCC appears easily achievable in humans, since other anti-tumor mAbs such as cetuximab and panitumumab achieve about 100-fold higher mean plasma peak levels in CRC patients upon recommended dosing [32,33]. With regard to potential toxicity/side effects, it must be considered that CD133 is not a tumor-exclusive antigen and, amongst others, expressed on healthy hematopoietic progenitor cells [34,35]. However, in our previous in vitro studies with 293C3-SDIE, no toxicity against healthy hematopoietic progenitor cells was observed, likely due to their profoundly lower CD133 antigen levels [14]. In addition, the first two clinical phase I studies evaluating CD133 targeting therapeutics—anti-CD133 CAR-T cells and DC-based CD133 vaccination—did not reveal any unbearable toxicity against healthy CD133 expressing cells [36,37]. Nevertheless, this issue and the question whether and how it is effective to target the CD133 positive cell fraction—potentially representing CSCs as reported in previous studies for CRC [38,39,40]—requires further elucidation. In any case, the results presented in this study demonstrate that 293C3-SDIE constitutes a promising novel option for CRC treatment, which we particularly envisage for elimination of residual disease after cytoreductive therapy.

## 4. Materials and Methods

### 4.1. Production, Purification, and Quality Control of Fc-Optimized Antibodies

293C3-SDIE and Iso-SDIE were produced as described previously [14]. In brief, plasmids for HC and LC were generated using the EndoFree Plasmid Maxi kit from Qiagen (Hilden, Germany) according to the manufacturer’s protocol. Antibodies were expressed in ExpiCHO cells (Gibco, Carlsbad, CA, USA) according to the manufacturer’s recommendations and purified by affinity (Mabselect; GE Healthcare, Chicago, IL, USA) and subsequent preparative size exclusion chromatography (HiLoad 16/60 Superdex 200; GE Healthcare, Chicago, IL, USA). Prior to use in functional experiments, mAbs were cleared from endotoxins using the Endotrap HD kit from Hyglos (Bernried, Germany). Ultimately, antibodies were run on analytical size exclusion columns (Superdex 200 Increase 10/300 GL; GE Healthcare; Chicago, IL, USA) and 4–12% gradient SDS-PAGE gels (Invitrogen; Carlsbad, CA, USA) using the gel filtration and Precision Plus standard from Bio-Rad (Hercules, CA, USA), respectively. 

### 4.2. Cells

B16F10-CD133 and B16F10-control cells were generated by transfecting B16F10 cells with pcDNA™3.1 based vectors coding for human CD133 (accession no. BC012089.1) or FLT3 (accession no. NM_004119.2) as control. Cells were cultivated in selection medium, i.e., Dulbecco’s Modified Eagle Medium (DMEM) containing 1 µg/mL G418 (Biochrom; Berlin, Germany).

The CRC cell lines Caco-2 and HCT-116 were from the German Collection of Microorganisms and Cell Cultures (Braunschweig, Germany) and HT-29 from the American Type Culture Collection (Manassas, VA, USA). The CRC cell lines SW-620 and COLO 205 were obtained internally at the University of Tuebingen. Authenticity was routinely determined by validating the respective immunophenotype described by the provider using flow cytometry after thawing, and cell lines were cultured for a maximum of 2 months prior to use in experiments. Contamination with mycoplasma was excluded by routine testing of all cultures every 3 months. All CRC cell lines were maintained in DMEM.

PBMC were isolated by density gradient centrifugation (Biocoll; Biochrom, Berlin, Germany) from thrombopheresis products of healthy volunteers and viably stored in liquid nitrogen. Prior to functional experiments, PBMC were cultured overnight in RPMI1640 for 18–24 h. 

All above-mentioned media contained Glutamax, 10% heat-inactivated fetal calf serum (Biochrom; Berlin, Germany), and 1% Penicillin/Streptomycin (Lonza; Verviers, Belgium). All cells were kept in a humidified atmosphere at 37 °C and 5% CO_2_.

### 4.3. Flow Cytometry

Flow cytometric analyses were performed using either fluorescently labeled or unlabeled mAbs followed by species-specific PE conjugates. Murine anti-human CD133 mAbs 293C3, AC133 and W6B3C1 were purchased from Miltenyi Biotec (Bergisch Gladbach, Germany). CD69-PE and CD107a-PE were from BD Pharmingen (San Diego, CA, USA), CD56-APC and CD14-PE/Cy7 from BioLegend (San Diego, CA, USA) and CD3-eFluor450 from eBioscience (San Diego, CA, USA). The goat anti-mouse PE conjugate was obtained from Dako (Glostrup, Denmark), the donkey anti-human PE conjugate was from Jackson ImmunoResearch (West Grove, PA, USA). The corresponding isotype controls were from BD Pharmingen (San Diego, CA, USA). Dead cells were excluded from analysis by 7-AAD (BioLegend; San Diego, CA, USA). Analysis was conducted using a FACS Canto II or FACS Fortessa (both BD Biosciences; Heidelberg, Germany). Specific fluorescence intensity (SFI) levels were calculated by dividing mean fluorescences obtained with a specific mAb by mean fluorescences obtained with the respective isotype control.

### 4.4. PCR Analysis

PCR analysis was performed as described previously [41]. In brief, total RNA was isolated using the High Pure RNA Isolation Kit (Roche, Mannheim, Germany) and transcribed into cDNA using FastGene Scriptase II (NIPPON Genetics Europe; Düren, Germany) according to the manufacturer’s instructions. CD133 primers were 5′-TGGGGCTGCTGTTTATTATTCT-3′ and 5′- TGCCACAAAACCATAGAAGATG-3′ [42]. Primer assays (QuantiTect Primer Assay) for 18S ribosomal RNA were from Qiagen (Hilden, Germany). Amplification of cDNA was performed using PerfeCTa SYBR Green FastMix (Quanta Biosciences; Beverly, MA, USA) on a LightCycler 480 (Roche, Basel, Switzerland) instrument. Relative CD133 mRNA expression—normalized to 18S rRNA—was calculated by the ΔΔ cycle-threshold (Ct) method.

### 4.5. Analysis of Direct mAb Effects on CRC Cell Viability

For analysis of direct mAb effects on CRC cell viability, CRC cell lines were seeded in white 96-well plates and treated with the indicated antibodies for 3 days. Subsequently, ATP levels as surrogate marker for live cells were determined using the CellTiterGlo assay from Promega (Madison, WI, USA) according to the manufacturer’s protocol. Cetuximab (Erbitux©) and panitumumab (Vectibix©) were from Eli Lilly (Indianapolis, IN, USA) and Amgen (Thousand Oaks, CA, USA). Staurosporin (Abcam; Cambridge, UK) was used as a positive control. Values depict means of technical triplicates with standard deviation.

### 4.6. Analysis of NK Cell Activation, Degranulation and Cytokine Secretion

PBMC of healthy donors were cultured with or without the indicated CRC cell lines at an effector/target (E:T) ratio of 2.5:1 in the presence or absence of 293C3-SDIE/Iso-SDIE (1 µg/mL). CD69 upregulation on NK cells (CD14-/CD56+/CD3- within PBMC fraction) after 24 h was analyzed by flow cytometry. For studies on NK cell degranulation, cells were cultured for 4 h in the presence of anti-CD107a-PE, BD GolgiStop and BD GolgiPlug (both BD Biosciences; Heidelberg, Germany). Subsequently, CD107a upregulation on NK cells was determined by flow cytometry. IFN-γ secretion into the supernatants was measured after 6 h by an enzyme-linked immunosorbent assay (ELISA) using the ELISA mAb set from Thermo Scientific (Rockford, USA) according to the manufacturer’s instructions. The KPL TMB Microwell Peroxidase Substrate System was from SeraCare Life Science (Milford, CT, USA) and the Streptavidin-Poly-HRP20 Conjugate from Fitzgerald Industries International (North Acton, MA, USA). If not indicated otherwise, IFN-γ values depict means of technical replicates with standard deviation.

### 4.7. Analysis of NK Cell Cytotoxicity 

Lysis of CRC cells by PBMC of healthy donors in the presence or absence of 293C3-SDIE/Iso-SDIE (1 µg/mL) was assessed by 2 h Europium based cytotoxicity assays as previously described [13]. Specific lysis was calculated as follows: 100× (experimental release—spontaneous release)/(maximum release—spontaneous release). If not indicated otherwise, values depict means of technical triplicates with standard deviation.

### 4.8. Statistics

Statistical analysis was performed with GraphPad Prism 8 (GraphPad Software, San Diego, CA, USA). The 95% confidence level was used, and p-values were calculated by one-way ANOVA and subsequent Tukey’s multiple comparison tests. Where indicated, statistically significantly different results (*p* < 0.05) between two groups are marked by “*”, and results not statistically different are marked by “ns”. 

## Figures and Tables

**Table 1:** Biological characteristics of the employed CRC cell lines.

Cell Line	Origin ^1^	MSI Status ^1^	KRAS ^1^	Relative CD133 mRNA ^2^	SFI 293C3 ^2^	SFI AC133 ^2^	SFI W6B3C1 ^2^
Caco-2	Primary tumor	MSS	wt	1.96 × 10^−3^	364.2	319.1	284.4
HCT-116	Primary tumor	MSI	G13D	1.99 × 10^−4^	76.9	62.1	70.7
HT-29	Primary tumor	MSS	wt	2.82 × 10^−4^	16.8	14.7	20.3
COLO 205	Metastasis	MSS	wt	3.33 × 10^−5^	3.0	2.3	3.5
SW-620	Metastasis	MSS	G12V	3.34 × 10^−4^	n.a.	n.a.	n.a.

^1^ Information on cell lines’ biological characteristics were derived from [24,25]. ^2^ Data were derived from Figure 1B,D. KRAS: Kirsten rat sarcoma viral oncogene; MSS/MSI: microsatellite stability/instability; n.a.: not applicable; SFI: specific fluorescence intensity.
